# The vitamin D receptor agonist EB1089 can exert its antiviral activity independently of the vitamin D receptor

**DOI:** 10.1371/journal.pone.0293010

**Published:** 2023-10-17

**Authors:** Janejira Jaratsittisin, Wannapa Sornjai, Thanathom Chailangkarn, Anan Jongkaewwattana, Duncan R. Smith

**Affiliations:** 1 Institute of Molecular Biosciences, Mahidol University, Salaya, Thailand; 2 Virology and Cell Technology Research Team, National Center of Genetic Engineering and Biotechnology (BIOTEC), National Science and Technology Development Agency (NSTDA), Pathum Thani, Thailand; Wayne State University School of Medicine, UNITED STATES

## Abstract

Vitamin D has been shown to have antiviral activity in a number of different systems. However, few studies have investigated whether the antiviral activity is exerted through the vitamin D receptor (VDR). In this study, we investigated whether the antiviral activity of a vitamin D receptor agonist (EB1089) towards dengue virus (DENV) was modulated by VDR. To undertake this, VDR was successively overexpressed, knocked down and retargeted through mutation of the nuclear localization signal. In no case was an effect seen on the level of the antiviral activity induced by EB1089, strongly indicating that the antiviral activity of EB1089 is not exerted through VDR. To further explore the antiviral activity of EB1089 in a more biologically relevant system, human neural progenitor cells were differentiated from induced pluripotent stem cells, and infected with Zika virus (ZIKV). EB1089 exerted a significant antiviral effect, reducing virus titers by some 2Log10. In support of the results seen with DENV, no expression of VDR at the protein level was observed. Collectively, these results show that the vitamin D receptor agonist EB1089 exerts its antiviral activity independently of VDR.

## Introduction

The most prevalent mosquito-borne flavivirus, dengue virus (DENV) is a major world health problem with an estimated 400 million people worldwide being infected each year [1]. Although the majority of infections are asymptomatic, DENV-infected patients can present with a wide range of symptoms ranging from a mild flu-like disease often with headache, fever, and rash to severe DENV infection, which can present with lethal complications such as plasma leakage, severe bleeding, and organ impairment [2]. Currently, the only licensed vaccine to protect against DENV infection, Dengvaxia, has encountered problems on account of a risk of severe disease when administered to DENV naïve individuals if they become subsequently naturally infected [3]. There are no specific anti-DENV drugs available, and most drug development investigations fail at the clinical trial phase [4]. A second important human pathogenic virus transmitted by mosquitoes in the genus Flavivirus is Zika virus (ZIKV). Starting from 2015 ZIKV emerged from Southeast Asia and rapidly became distributed over much of the tropical and subtropical world [5]. As with DENV infection the majority of infections are asymptomatic, but ZIKV-infected patients can present with symptoms similar to DENV infection and the disease is largely mild and self-limiting [6]. However, when a pregnant woman becomes infected in the first or second trimester, the virus can cross the placenta to infect the developing fetus, resulting in significant developmental abnormalities including microcephaly [7].

During the COVID-19 pandemic caused by the severe acute respiratory syndrome coronavirus 2 (SARS-CoV-2), vitamin D has been recognized as a valuable supplement exhibiting antiviral action against SARS-CoV2, with a study showing a correlation between serum vitamin D concentration and the risk of virus infection, with higher levels of SARS-CoV-2 being found in patients with a low level of serum vitamin D [8]. More recently a randomized controlled trial has shown positive benefits in vitamin D supplementation to hospitalized COVID-19 patients [9].

Vitamin D is a secosteroid hormone generated by UVB exposure of the skin and from some dietary sources. The active vitamin D or 1,25-dihydroxyvitamin D3 (calcitriol) requires activation via the cytochrome P450 (CYP) enzyme [10]. The intermediate vitamin D, 25-hydroxyvitamin D3, is hydrolyzed via the 25-hydroxylase enzyme encoded by CYP2R1 in the liver [11]. Subsequently, another hydroxylation via CYP27B1 encoding 1α-hydroxylase in the kidney synthesizes the biologically active form of 1,25-dihydroxy vitamin D3 (1,25D) or calcitriol [12]. Vitamin D catabolism is strongly induced by 1,25D via the 24-hydroxylase enzyme (CYP24A1) as a negative feedback loop [10]. Once in a cell, 1,25D binds to its nuclear receptor super-family vitamin D receptor (VDR) and induces the heterodimerization with retinoid X receptor (RXR). This regulatory complex translocates into the nucleus and binds with the specific nucleotide DNA sequences containing a vitamin D responsive element (VDRE) [13]. Subsequently, the DNA-bound VDR/RXR heterodimer recruits several regulatory proteins which control histone modifications, chromatin remodeling, RNA polymerase III binding, and transcription initiation [14].

The classic vitamin D function involves regulating calcium and phosphate homeostasis, which is mediated through activation of VDR resulting in the expression of numerous genes. However, VDR and related-enzymes can be found in many non-calcium regulating cells such as fibroblasts, keratocytes, cardiovascular cells, and immune cells [15–17] and vitamin D and VDR have been shown to modulate a number of other essential biological processes, including cell growth/ proliferation, immunomodulation, as well as having anti-cancer and antiviral activity [18–20]. The common limitation of 1,25D when used in treatment is hypercalcemia, and the vitamin D structure is unstable and easily converted to an inactive form by CYP24A1. The vitamin D analog, EB1089 or seocalcitriol has been modified at the side chain but still exhibits a high VDR binding affinity, with less hypercalcemia side effects [21]. This vitamin D analog has been shown to inhibit cell proliferation and metastasis of several cancer types including breast, pancreatic and colorectal cancer and EB1089 also induced apoptosis and autophagy cell death pathways [22–25]. Furthermore, the antiviral action of EB1089 has been demonstrated in DENV infection, whereby EB1089 reduced viral production and mimicked the gene regulatory effects of 1,25D [26].

Besides the classical vitamin D pathway, in an alternative pathway 1,25-dihydroxy vitamin D3 directly interacts with membrane-bound proteins or intracellular targets. This rapid and non-genomic response to vitamin D is mediated through the protein disulfide isomerase family A member 3 (PDIA3), also known as ERp57 or MARRS (membrane-associated rapid response to steroid). PDIA3 or membrane VDR (VDRm) interacts with caveolin 1 (CAV1) in the caveolae [27–29] and responds to vitamin D via the activation of membrane signaling cascades, including the protein kinase C (PKC), phospholipase A2 (PLA2), phosphatidylinositol 3-kinase (PI3K), and mitogen-activated protein kinase (MAPK) pathways [30–33]. Furthermore, PDIA3 is proposed to directly interact with the target proteins involved in antiviral gene transcription, such as the cathelicidin antimicrobial peptide (CAMP), NF-κB, and STAT-1,2 [28,34].

In the past decade, the antiviral activity of vitamin D has been highlighted for several viruses including the human immunodeficiency virus (HIV, recently reviewed in [35]), influenza [36], severe acute respiratory syndrome coronavirus 2 (SARS-CoV-2) [8,9], hepatitis C virus (HCV) [37], DENV [26,38–45], and one study previously has shown activity of a VDR agonist against ZIKV [26]. Markedly however, most studies have not evaluated whether vitamin D is exerting an antiviral effect through the classic VDR mediated pathway, and interestingly, one study undertaken with HCV determined that vitamin D exerted its antiviral effect independently of VDR [46]. This study sought to determine whether a vitamin D receptor agonist (EB1089) exerted its antiviral activity through, or independent of VDR. We additionally sought to determine if EB1089 was able to exert an antiviral effect in ZIKV-infected human neural progenitor cells derived from induced pluripotent stem cells (iPSCs) as a more biologically relevant model system.

## Materials and methods

### Cells and virus

HEK293T/17 (ATCC No. CRL-11268) cells were maintained in Dulbecco’s Modified Eagle Medium (DMEM; Thermo Fisher Scientific, Waltham, MA) supplemented with 10% heat-inactivated fetal bovine serum (FBS; Thermo Fisher Scientific, Waltham, MA) at 5% CO_2_, 37°C. LLC-MK2 (ATCC CCL-7) and Vero (ATCC CCL-81) cells were cultured at 37°C with 5% CO_2_ in Dulbecco’s modified Eagle’s medium (DMEM, GIBCO, Invitrogen) supplemented with 5% FBS and 100 units/ml of penicillin/streptomycin. Human induced pluripotent stem cells (hiPSCs) MUSli011-A [47] were maintained in mTeSR medium (Stemcell Technologies, Vancouver, Canada) on Matrigel (Cat. No. 354234; Corning, NY) -coated 6-well plate at 5% CO_2_, 37°C.

DENV 2 strain 16681 and ZIKV Thai strain SV0010/15 were propagated in the Aedes albopictus cell line C6/36 (ATCC No.CRL-1660) as described previously [48]. The supernatants were collected, centrifuged to remove cell debris before storage at -80°C until use. The virus stocks were verified by DNA sequencing and the virus titer was determined by plaque assay in LLC-MK2 (ATCC No.CCL-7) for DENV or Vero cells (ATCC No. CCL-81) for ZIKV. All work with live viruses was undertaken in a BSL 2 level laboratory, after Institutional Biosafety Committee approval.

### Plaque assay

Infectious virus titers were quantitated by plaque assay. Either LLC-MK2 cells (for DENV) or Vero cells (for ZIKV) were cultured in a 6-well plate with DMEM supplemented with 5% fetal bovine serum for 24 h at 37°C and 5% CO_2_. Briefly, stock viruses or experimental supernatants were 10-fold serially diluted with BA-1 virus diluent (1X medium 199/Earle’s balanced salts, 0.05 M Tris-HCl pH 7.6), 1% serum albumin, 0.075% NaHCO_3_, and 100U of penicillin-streptomycin per mL. Then, the cells were infected with 200 μL of the diluted virus and gently rocked every 10 min for 2 h at 37°C. Subsequently, the infected cells were gently overlaid with 4 mL/well of 1X nutrient solution with 0.8% (W/V) Seakem LE agarose (Merck KGaA, Darmstadt, Germany) for DENV and 1.2% methylcellulose (Merck KGaA, Darmstadt, Germany) in DMEM supplemented with 2% FBS for ZIKV. Six days post-infection, the virus was again overlaid with a nutrient agarose containing 0.4% neutral red and plates were further incubated for another 24 h at 37°C, with 5% CO_2_. For ZIKV, the overlay medium was removed on day 7 of infection. The infected cells were subsequently fixed with 3.7% formaldehyde (Merck KGaA, Darmstadt, Germany) in 1X-PBS for an hour at room temperature. Then, the fixing solution was discarded and plates were washed with water before staining with 1% crystal violet (Merck KGaA, Darmstadt, Germany) in 20% ethanol. Finally, the plaques were counted and calculated as a PFL/mL unit virus titer. All plaque assays were undertaken on independent biological triplicates with duplicate plaque assays.

### VDR overexpression

The VDR gene coding sequence was amplified by RT-PCR from RNA from HEK293T/17 cells using specific primers with restriction enzyme sequences added (pcDNA_VDR_F: 5’- AATAAGCTTGCCACCATGGAGGCAATGGCGG-3’, pcDNA_VDR_R: 5’- GGTGGATCCTCAGGAGATCTCATTGC -3’). After amplification and electrophoresis through an agarose gel the target band was cut and purified with the FavorPrep GEL/PCR Purification Kit (Favorgen, Pingtung, Taiwan). The purified cDNA was ligated into the pcDNA3.1+ eukaryotic expression plasmid (Invitrogen, Waltham, MA) using ligase enzyme at room temperature overnight. Then, the recombinant plasmid was transformed into Escherichia coli DH5α by the heat shock method and transformants were grown on an LB agar plate containing ampicillin at 37°C for 16 h. The correct clones were confirmed by colony PCR, restriction enzyme digestion, and DNA sequencing. The pcDNA_VDR transfection reaction was optimized, and the protein level was determined using western blot assay. HEK293T/17 cells were cultured on a 12-well plate and transfected with the optimized condition of pcDNA_VDR. After 24 h post-transfection, the transfected cells were subsequently infected with DENV 2 at MOI 5 for 2 h, followed by treatment with 20 μM of the vitamin D analog EB1089 (Cat. No. 3993; Tocris Bioscience, Bristol, UK). A 0.04% concentration of DMSO was used as a diluent control of the EB1089 treatment. Then, the supernatant and cells were collected at 24 h post-infection and antiviral activity was determined, including the levels of infectious virus by plaque assay and viral protein expression by western blotting. Moreover, vitamin D-related gene transcription levels were determined.

### VDR knockdown using siRNA

HEK293T/17 cells (with no prior transfection) were cultured in a 24-well plate at 60% cell confluency in DMEM supplemented with 10% FBS. Then, the cells were incubated with a total volume of 60 μl of the transfection mixture, composed of 20 ρmole of validated siRNA VDR (4010, Thermo Fisher Scientific Inc., Waltham, MA), and Lipofectamine™ RNAiMAX Transfection Reagent (Thermo Fisher Scientific Inc., Waltham, MA). GFP siRNA (Silencer GFP (eGFP) siRNA; U55761, Ambion, Austin, TX) was selected as a non-targeting siRNA. After 48 h of transfection, the knockdown of expression of VDR was confirmed at the protein expression level. Subsequently, the VDR knockdown cells were infected with DENV 2 for 2 h, followed by treatment with 20 μM of v EB1089 for 24 h, in parallel with the siGFP transfected cells. A 0.04% concentration of DMSO was used as a diluent control of the EB1089 treatment. The effect of VDR knockdown on the antiviral action of EB1089 treatment was determined including the levels of infectious virus by plaque assay and viral protein expression by western blotting. Moreover, vitamin D-related gene transcription levels were determined.

### Mutation of nuclear localization sequences of VDR

Firstly, the VDR protein was tagged with GFP to allow observation of the cellular location of the constructs under a confocal microscope. In brief, the VDR gene without a stop codon was amplified with specific primers (F_VDR_hindIII: 5’-AATAAGCTTGCCACCATGGAGG-3’, R_VDRnostop_KpnI: 5’-GGGGTACCCGGAGATCTCATTGCCAAACACTTCG-3’). Then, this gene fragment was inserted at the N-terminal of the GFP sequence using restriction enzyme digestion, ligation, and transformation, respectively. The sequences were confirmed by DNA sequencing. Then, the expression levels were determined after the transfection of 3 μg of pEGFPN2_VDR into HEK293T/17 cells for 24 h.

After obtaining the pEGFPN2_VDR expression vector, site-directed mutagenesis was performed at the nuclear localization signal (NLS) in the DND-binding domain of the VDR gene. The mutagenesis primers were designed to substitute amino acid sequences 49RR/KR54 for 49AA/AA54 [49]. First, the amplification was performed twice between the VDR cloning primer and mutagenesis primer (F_VDRmut: GGCTTCTTCGCCGCAAGCATGGCCGCCAAGGCACTATTCACCTGCCCCTTC, R_VDRmut: GTGCCTTGGCGGCCATGCTTGCGGCGAAGAAGCCTTTGCAGCCTTCACAGG). Then, these two fragments were used as the templates for overlapping PCR to get the whole piece of VDR gene containing the mutation. Next, the PCR products were digested with specific restriction enzymes, ligated, and transformed. Finally, the mutation at the nuclear localization signal of VDR was confirmed by DNA sequencing.

The HEK293T/17 cells were cultured on coverslips, and then cells were transfected with 3 μg of pEGFFPN2_VDRmut in parallel with pEGFFPN2_VDR (wild type) or mock transfected (No DNA). Firstly, the coverslips were collected, and an immunofluorescence assay was performed to observe the GFP-tagged VDR protein expression after 24 and 48 h post-transfection. To determine the antiviral action of EB1089 HEK293T/17 cells on coverslips were transfected with 3 μg pEGFPN2_VDR or pEGFFPN2_VDRmut or mock transfected (no DNA) for 24 h, and then the transfection mixture was discarded. The transfected cells were infected with DENV 2 at MOI 5 for 2 h, after which cells were treated with 20 μM of EB1089. A 0.04% concentration of DMSO was used as a diluent control of the EB1089 treatment. After a further 24 h of incubation, the supernatant and coverslips of treated cells were collected, and antiviral activity was determined via plaque assay of supernatants and examination of DENV E protein expression levels under a confocal microscope.

### Western blot assay

To obtain total proteins, cells were collected and lysed in RIPA lysis buffer containing a protease inhibitor cocktail (Merck KGaA, Darmstadt, Germany). Lysates were centrifuged at 12,000 g at 4°C for 15 min after which the protein concentration was determined by the Bradford assay (Bio-Rad, San Francisco, CA). A total of 30 μg total protein was separated on 12% sodium dodecyl sulfate-polyacrylamide gels by electrophoresis (SDS-PAGE) gels and then the separated proteins were transferred to nitrocellulose membranes (Whatman GmbH, Germany). Non-specific proteins were blocked with 5% skim milk in TBST (1X Tris-buffered saline containing 0.05% Tween-20) at room temperature, and subsequently, membranes were incubated with a primary antibody in 5% skim milk TBST at 4°C overnight. The primary antibodies used were a mouse monoclonal anti-dengue serotype 1–4 antibody (MA1-27093; Thermo Fisher Scientific Inc., Waltham, MA), a mouse monoclonal anti-dengue NS5 antibody (GTX629446, GeneTex, Irvine, CA), a mouse monoclonal anti-VDR (D-6) antibody (sc-13133; Santa Cruz Biotechnology Inc., Dallas, TX), a rabbit polyclonal anti-Zika virus envelope protein antibody (GTX133314; GeneTex, Inc, Irvine, CA), and a mouse monoclonal anti-GAPDH antibody (sc-32233; Santa Cruz Biotechnology Inc., Dallas, TX). After incubation, membranes were washed three times with TBST and then incubated with an HRP-conjugated polyclonal goat anti-mouse IgG antibody (31430; Thermo Fisher Scientific Inc., Waltham, MA) or a HRP-conjugated polyclonal goat anti-rabbit IgG antibody (31460; Thermo Fisher Scientific Inc., Waltham, MA) in 5% skim milk TBST for 1 h at room temperature. Again, the membranes were washed three times with TBST and the signal was developed by using the ECL Plus Western Blotting Analysis kit (Amersham Pharmacia Biotech, Piscataway, NJ) and visualized using X-Ray films. The bands were quantified using the ImageJ analysis software and normalized with the GAPDH loading control. Experiments were performed independently in triplicate. Details of primary and secondary antibodies and dilutions used can be found in S1 and S2 Tables, respectively.

### Immunofluorescence assay

HEK293T/17 cells grown on glass coverslips were mock-or DENV infected followed by treatment with 20 μM EB1089 for 24 h. After that time, the glass coverslips were collected and fixed with 4% paraformaldehyde for 20 minutes. Then, the cells were washed twice with 1X PBS and non-specific signals were blocked by incubation with 10% bovine serum in 1X PBS. After washing twice with 0.03% Triton X-100 in 1XPBS/IFA, the cells were permeabilized by incubation with 0.3% Triton X-100 in 1XPBS/IFA. Viral proteins were detected with a mouse monoclonal anti-dengue serotype 1–4 antibody (MA1-27093; Thermo Fisher Scientific Inc., Waltham, MA) or a rabbit polyclonal ZIKV envelope protein antibody (GTX133314; GeneTex, Inc, Irvine, CA). In addition, the neural progenitor cells were confirmed by staining with specific protein markers, including an anti-human PAX6 antibody (Miltenyi Biotec, Bergisch Gladbach, Germany), an anti-human SOX1 antibody (R&D Systems, Minneapolis, MN), an anti-human Nestin antibody, Clone 10C2 (Stemcell Technologies, Vancouver, Canada), and a recombinant Anti-Musashi-1 / Msi1 antibody [EP1302] (Abcam, Cambridge, United Kingdom). Subsequently, the appropriate secondary antibodies were chosen including a donkey anti-mouse Alexa 488, a donkey anti-goat Alexa 568, a donkey anti-rabbit Alexa 647 and a goat anti-human Alexa 647 (Thermo Fisher Scientific Inc., Waltham, MA), and the nucleus was stained with DAPI, FluoroPure grade (Thermo Fisher Scientific Inc., Waltham, MA). Finally, the coverslips were mounted onto glass slides using Prolong Gold anti-fade reagent (Invitrogen, Waltham, MA) before visualization under a confocal microscope, LSM 800w Airy scan (‎ZEISS, Oberkochen‎, Germany). Details of primary and secondary antibodies and dilutions used can be found in S1 and S2 Tables, respectively.

### VDR-mediated gene expression

VDR-mediated gene expression analysis was undertaken essentially as described previously [26]. Briefly, cells were lysed and RNA was extracted using Trizol (Thermo Fisher Scientific Inc., Waltham, MA) according to the manufacturer’s recommendations. cDNA was synthesized using oligo(dT) primers and RevertAid reverse transcriptase (Thermo Fisher Scientific Inc., Waltham, MA). The levels of gene expression were determined using qRT-PCR with KAPA SYBR FAST qPCR Kit 2X Master MIX (Kapa Biosystem Inc., Waltham, MA) with specific primers, including VDR-F: 5′-TGCTATGACCTGTGAAGGCTG-3′, VDR-R: 5′-AGTGGCGTCGGTTGTCCTT-3′, CYP24A1-F: 5′-CAGCGAACTGAACAAATGGTCG-3′, CYP24A-R 5′-TCTCTTCTCATACAACACGAGGCAG-3′, CYP27B1-F: 5′-GAATTGCAAATGGCTTTGGCCCAG -3′, and CYP27B1-R 5′-CTGTAGGTTGATGCTCCTTTCAGG -3′. The relative expression was calculated by the normalization against the β-actin gene followed by uninfected cells with mock transfection or untreated condition. According to the following equation: ΔΔCt = ΔCt (sample)—ΔCt (mock). Experiments were performed independently in triplicate with triplicate qRT-PCR.

### Neural progenitor differentiation

Neural progenitor cells (NPCs) were differentiated from human induced pluripotent stem cells derived from peripheral blood T lymphocytes of a healthy individual [47]. The NPC differentiation was performed via embryoid body formation, following the technical manual of the STEMdiff SMADi Neural Induction Kit (STEMCELL Technologies Inc., Vancouver, Canada). Briefly, hiPSC colonies were enzymatically dissociated and 3 x 106 cells were plated in a single well of AggreWell 800 24-well plates (STEMCELL Technologies Inc., Vancouver, Canada) with STEMdiff Neural Induction Medium (STEMCELL Technologies Inc., Vancouver, Canada) to generated embryoid bodies (EBs). A total of 75% of the media volume was gently changed for fresh media daily for 5 days. Next, the EBs were collected and re-plated into a single well of a Matrigel-coated 6-well plate. Then a daily full-medium change was performed using warm STEMdiff neural induction medium for another 6 days. Finally, the signature neural rosette formation was selected using the STEMdiff neural rosette selection reagent and incubated for 1 h at 37°C. Consequently, the detached neural rosettes were collected and transferred into a single well of a Matrigel-coated 6-well plate. The media was completely changed daily for another 5 days. Finally, the neural progenitor cells were sub-passaged using accutase (STEMCELL Technologies Inc., Vancouver, Canada) onto Matrigel-coated plates or cell culture flasks with the NPC media supplemented with freshly prepared 20 ng/ml basic fibroblast growth factor (bFGF) (STEMCELL Technologies Inc., Vancouver, Canada). The medium was composed of Dulbecco’s Modified Eagle Medium/Nutrient Mixture F-12: DMEM/F-12 (Thermo Fisher Scientific Inc., Waltham, MA), GlutaMAX Supplement (Thermo Fisher Scientific Inc., Waltham, MA), B-27 Supplement (50X) (Thermo Fisher Scientific Inc., Waltham, MA), N-2 Supplement (100X) (Thermo Fisher Scientific Inc., Waltham, MA), and EmbryoMax Penicillin-Streptomycin Solution, 100X (Merck, Darmstadt, Germany). The NPC characteristics were confirmed by neural progenitor marker staining using an immunofluorescence assay before experimental use.

### The antiviral activity of EB1089 in hiPSCs-derived NPCs

First, the cytotoxicity of EB1089 was evaluated in the NPCs prior to selecting the optimal concentration to determine its antiviral action. NPCs cultured in Matrigel-coated 96-well plates were either untreated, or incubated with EB1089 diluted with NPC medium to various concentrations in parallel with a DMSO vehicle control in a final volume of 100 uL per well. After 24 h of treatment, MTT dye (Thermo Fisher Scientific Inc., Waltham, MA) was added into the wells and samples were incubated at 37°C for one hour, followed by an hour incubation to dissolve the formazan precipitate with DMSO. Then the optical density was measured at 570 nm using an EZ read 2000 microplate reader (Biochrome, Cambridge, United Kingdom). The cell viability was calculated as the percentage compared with the untreated cells and the half-maximal cytotoxicity concentration or CC50 was determined using the freeware ED50plus (v1.0) software (https://sciencegateway.org/protocols/cellbio/drug/data/ed50v10.xls).

To determine the antiviral activity of EB1089 in NPCs, cells were cultured on Matrigel-coated coverslips in 24-well plates at a density of 5X10^4^ cells/cm^2^. The cells were incubated with ZIKV (SV0010/15) with MOI 20 for 2 h after which the inoculum was removed. Then the infected cells were treated with 10 μM of EB1089, which was determined from the CC50 of 20.48 μM of EB1089 on NPCs. At 24 h post-treatment, the supernatant was collected to determine the levels of infectious virions via plaque assay, while cells were evaluated using western blot and immunofluorescent assay.

### Statistical analysis

All experiments were performed independently in triplicate with a duplicate plaque assay. The results are displayed as mean ± SEM. The data were subsequently analyzed using the GraphPad Prism program (GraphPad Software Inc., San Diego, CA). Analysis was performed using the independent t-test of PASW Statistics 18.0.0 (SPSS Inc., Chicago, IL) and the statistical significance is shown with p-values of < 0.05*, < 0.01** and< 0.001***.

## Results

### Effect of overexpression of VDR on the antiviral activity of EB1089

**Fig 1:**
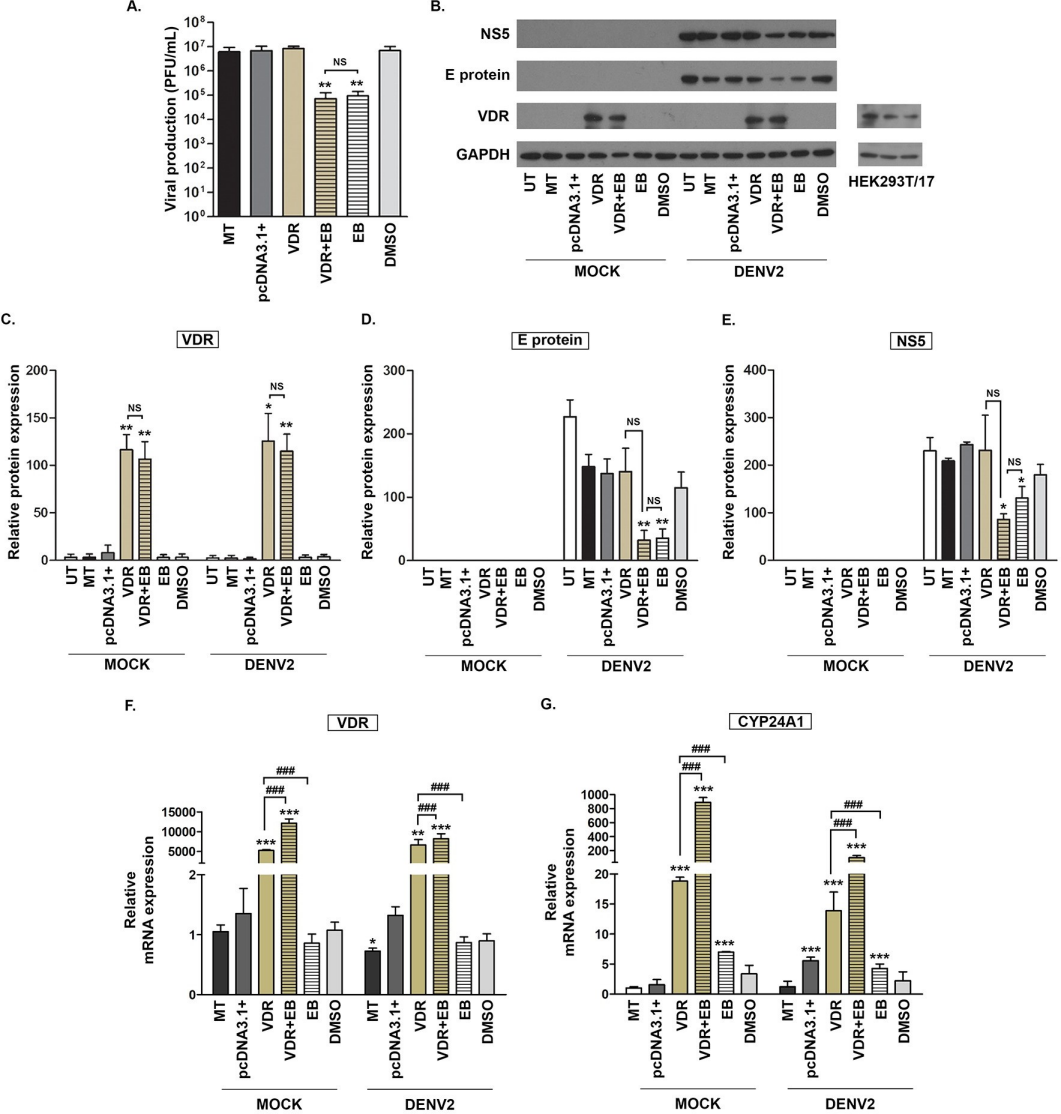
Effect of overexpression of VDR on the antiviral activity of EB1089. HEK293T/17 cells were either untreated, mock transfected, or transfected with pcDNA_VDR or an empty plasmid pcDNA3.1+ as a transfection control. After 24 h post-transfection, the media was discarded and cells were either mock infected or infected with DENV 2 at MOI 5 for 2 h. Then, the cells were incubated with normal media, or with media containing 20 μM of EB1089, or with a DMSO vehicle control for 24 h, after which cells and supernatants were collected for analysis. (A) The infectious virion production in the supernatant was evaluated by plaque assay, while (B) levels of expression of VDR, E, NS5 and GAPDH were determined by western blotting protein, and band intensities of (C) VDR, (D) E protein, and (E) NS5 were analyzed using the image J program and normalized to GAPDH. (F, G) The levels of gene expression for (F) VDR and (G) CYP24A1 were determined by real-time PCR. All experiments were conducted independently in triplicate with a duplicate plaque assay. p-value; *< 0.05, **< 0.01, ***< 0.001 for significance compared with mock transfection, and # significance compared between each additional untreated/treated condition. UT, untreated cells; MT, mock transfection; pcDNA3.1+, empty vector transfection; VDR, transfection with pcDNA_VDR; VDR+EB, transfection with pcDNA_VDR and treatment with EB1089; EB, treatment with EB1089; DMSO: Vehicle control. Cropped images of western blots are shown, and full un-cropped images can be found in the supplementary materials.

**Fig 2:**
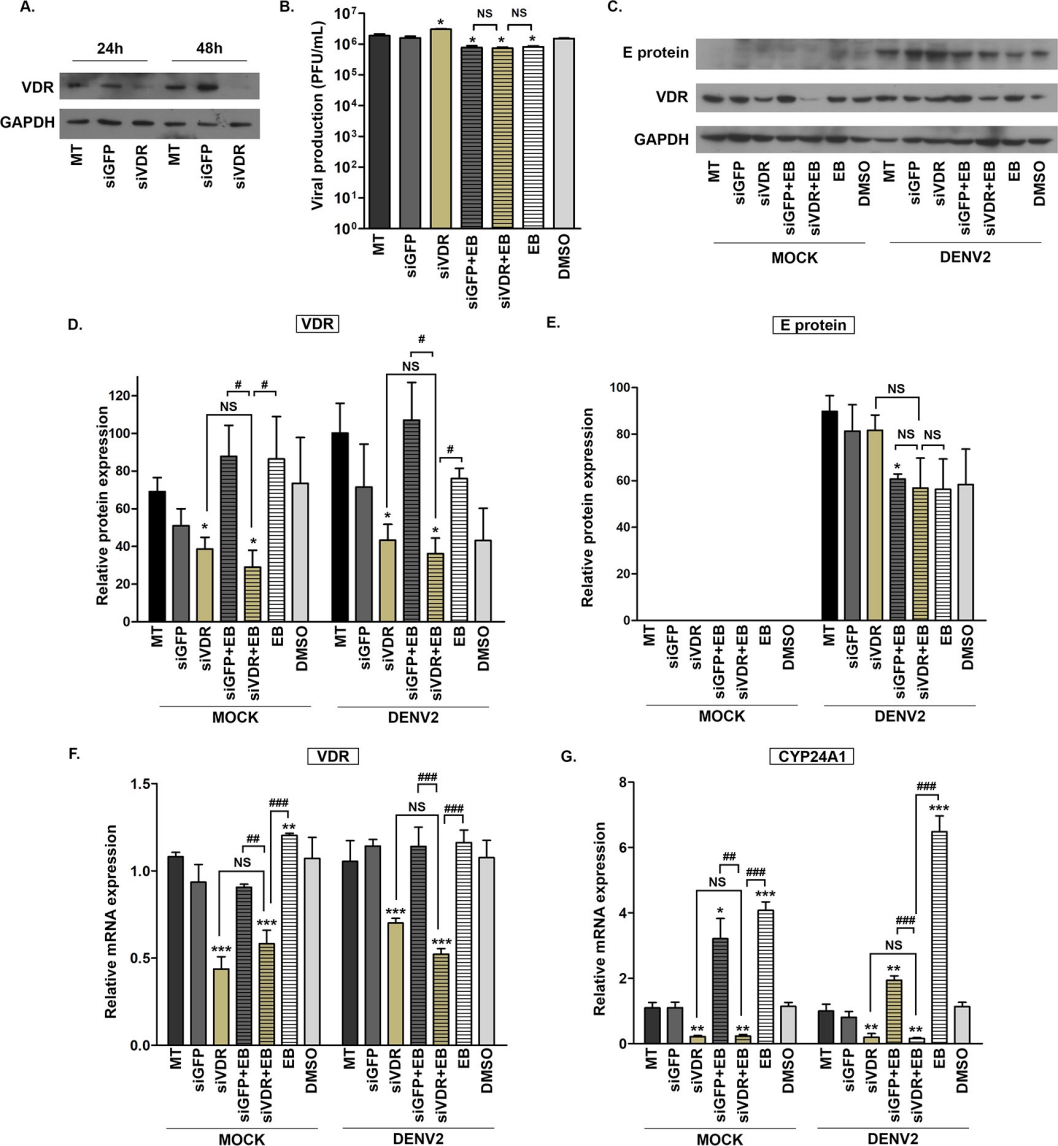
Effect of knockdown of VDR expression on the antiviral activity of EB1089. (A) HEK293T/17 cells were either not transfected, or were transfected with a validated siVDR or a siGFP for 24 and 48 h after which down-regulation of VDR protein expression was evaluated via western blot assay. Subsequently, HEK293T/17 cells were either not transfected, or were transfected with a validated siVDR or siGFP for 48 h after which cells were mock infected or infected with DENV 2 at MOI 5 for 2 h, after which the media was replaced with standard media, or standard media containing 20 μM of EB1089 or DMSO vehicle control. At 24 h post-infection, the supernatant and the infected cells were collected for analysis by (B) plaque assay to determine supernatant virus titer, or (C) western blot analysis of VDR, E protein and GAPDH expression. The relative protein expression levels were analyzed using Image J and signals for (D) VDR and (E) E protein was determined and normalized with GAPDH. (F, G) The levels of gene expression for (F) VDR and (G) CYP24A1 were determined by real-time PCR. All experiments were conducted independently in triplicate with a duplicate plaque assay. p-value; *< 0.05, **< 0.01, ***< 0.001 for significance compared with mock transfection, and # significance compared between each additional untreated/treated condition. MT, mock transfection; siGFP, cells transfected with siGFP; siVDR, cells treated with siVDR; siGFP+EB, cell transfected with siGFP and treated with EB1089; siVDR+EB, cells transfected with siVDR and treated with EB1089; EB, treatment with EB1089; DMSO, DMSO vehicle control. Cropped images of western blots are shown, and full uncropped images can be found in the supplementary materials.

To understand how vitamin D receptor agonists exert their antiviral activity, expression of VDR was upregulated. To achieve this, HEK293T/17 cells were plated into 12-well plates and either untreated, or mock transfected, or transfected with empty plasmid or transfected with pcDNA_VDR for 24 h. The untreated, transfected and mock-transfected cells were then mock infected or infected with DENV 2 at MOI 5 for 2 h, after which the media was replaced with media containing 20 μM of EB1089 or with vehicle control. At 24 h post-treatment, the supernatant and cells were collected to determine virus titer and viral protein production, respectively. To avoid overgrown cells, 24 h-post infection was selected as the time point for evaluation. The plaque assay showed that overexpression of VDR alone had no effect on virus titer as compared to controls (either mock transfection or DMSO vehicle control), while treatment with EB1089 alone reduced virus titer by some 2Log10 (Fig 1A). Markedly, overexpression of VDR combined with EB1089 treatment showed a similar 2Log10 reduction in virus titer as found with EB1089 treatment alone (Fig 1A). To look at effects on viral protein expression, some of the harvested cells were used to prepare proteins, which were subjected to PAGE and subsequent transfer to filters. The expression of DENV E protein and NS5 were investigated, together with cellular VDR and GAPDH expression by western blotting. The results showed that VDR expression was markedly increased in cells transfected with the VDR expression vector (Fig 1B and 1C), and that reduction of expression of DENV protein was limited to cells that had been treated with EB1089. Markedly, again no significant difference in the degree of reduction in DENV protein expression was seen between cells treated with EB1089 alone, and cells transfected with the VDR construct and then treated with EB1089 (Fig 1B, 1D and 1E). We note that over-expression of VDR resulted in such a high signal that endogenous VDR could not be seen in western blots. A separate probing of untransfected HEK293T/17 cells however clearly showed that endogenous VDR was expressed in these cells (Fig 2B).

The remaining portion of the harvested cells was used to prepare RNA to investigate the gene regulatory effects of EB1089 using qRT-PCR. Unsurprisingly, levels of expression of the VDR transcript were significantly raised in cells transfected with the VDR construct (Fig 1F). A significant upregulation was additionally observed between VDR transfected and VDR transfected/EB1089 treated conditions in both mock infected cells and DENV 2 infected cells (Fig 1F). One downstream gene whose expression is mediated by VDR is CYP24A1, which is part of the vitamin D degradation pathway [10]. Analysis of this gene expression in mock infected cells revealed a substantial rise in CYP24A1 levels with VDR transfection, EB1089 treatment alone, and even greater levels when both VDR transfection and EB1089 treatment were combined (Fig 1G). This result shows convincingly that the VDR construct used in this series of experiments was biologically active, and that over-expression could modulate downstream transcriptional signaling. In DENV infected cells again minor (but significant) changes were seen in CYP24A1 expression in response to control plasmid transfection and EB treatment, but these changes were minor in comparison to the VDR transfection, and particularly the VDR transfection and EB1089 treatment (Fig 1G). In summary, no differences were seen in antiviral activity between cells treated with EB1089 with or without increased VDR expression.

### Effect of knockdown of VDR expression on the antiviral activity of EB1089

To further understand the antiviral action of VDR agonists in relationship to VDR, siRNA was used to knock down the expression of VDR before treatment with EB1089. HEK293T/17 cells were therefore transfected with a validated VDR siRNA in parallel with a non-target GFP siRNA transfection control and non-siRNA treated cells for 48 h, after which the cells were collected to determine the siRNA knockdown efficiency. The results showed the successful knockdown of VDR protein expression as compared to siGFP or non-siRNA treated cells (Fig 2A). Subsequently, the transfected cells were infected with DENV and incubated with EB1089 for 24 h, after which the supernatant was collected and the cells were harvested and split into two for protein and RNA extraction. The results of plaque assay demonstrated that EB1089 treatment alone or with either siVDR or non-targeted siGFP transfected cells showed similar reduction in virus titer with approximately 0.5Log10 when compared to non-transfection control (Fig 2B). Analysis of VDR protein expression showed that by the time point of collection, siVDR transfection significantly reduced VDR levels in both mock and DENV infected cells. Furthermore, the presence of EB1089 in VDR silenced cells was not able to raise the VDR protein levels, contrary to the treatment with EB1089 alone or in siGFP transfected cells (Fig 2C and 2D). The antiviral effect of EB1089 on the reduction of DENV E protein expression was observed in both VDR silenced cells and in non-targeted siGFP transfected cell with the same magnitude as the non-transfected cells (Fig 2C and 2E). Additionally, the analysis of VDR mRNA levels demonstrated similar results to protein levels, where siVDR transfection efficiently disrupted mRNA expression in both mock-infected and DENV infected cells (Fig 2F). Consequently, these effects were further influenced on the gene regulatory activity of VDR on CYP24A1 gene which was indicated by the significant aberrant activation of CYP24A1 in VDR suppressed cells (Fig 2G). Additionally, the presence of EB1089 on siVDR transfected cells still was not able to induce the CYP24A1 gene transcript compared with the treatment of EB1089 alone or on the siGFP transfected cells (Fig 2G). In summary, similar antiviral activity as assessed by the inhibition of virus titer and viral protein production was observed between cells treated with EB1089, with or without knockdown of VDR expression.

### Effect of inhibition of nuclear translocation of VDR on the antiviral activity of EB1089

**Fig 3:**
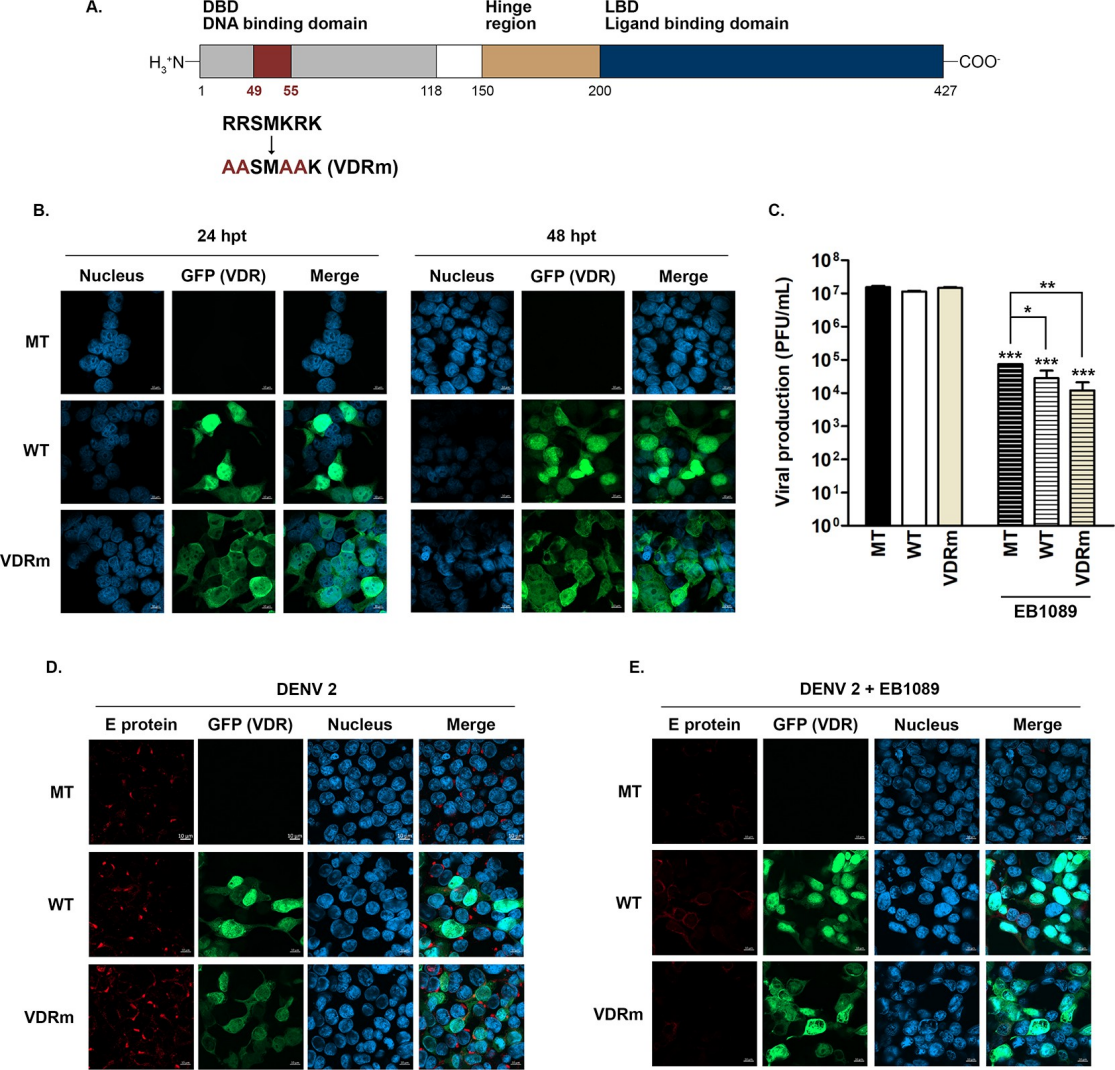
Effect of inhibition of nuclear translocation of VDR on the antiviral activity of EB1089. (A) The nuclear localization sequences of VDR were mutated at amino acids R49A, R50A, K53A, and R54A by site-directed mutagenesis as shown in the schematic. (B) HEK293T/17 cells were mock transfected or transfected with GFP-tagged mutated VDR (VDRm) or a GFP-tagged wild-type VDR (WT) for 24 h. The pattern of expression of the constructs was investigated at 24 and 48 h post-transfection, compared with mock-transfected cells (MT) under a confocal microscope. (C-E) HEK293T/17 cells were mock transfected or transfected with GFP-tagged mutated VDR (VDRm) or a GFP-tagged wild-type VDR (WT) for 24 h after which they were infected with DENV 2 at MOI 5, after which the media was replaced with normal media or media containing20 μM EB1089. At 24 h post-infection, cells and supernatant were collected and (C) virus titer in the supernatant was quantitated by plaque assay; (D, E) cells were stained with an antibody directed against DENV E protein (red) and with DAPI (blue) and examined under a confocal microscope. GFP (green) in constructs was also examined. Experiments were undertaken independently in triplicate, and representative images are shown. Plaque assay was undertaken in duplicate for each of the triplicates. p-value; *< 0.05, **< 0.01, ***< 0.001 for significance.

The VDR non-mediated antiviral action of vitamin D analog EB1089 was further investigated by inhibiting the nuclear translocation of VDR. The nuclear localization sequences (NLSs) in the DNA-binding domain of the VDR gene were mutated via site-directed mutagenesis at the amino acid sequences 49 to 55 from RRSMKRK to AASMAAK as shown in Fig 3A. HEK293T/17 cells were transfected with the VDR mutated gene tagged with GFP (VDRm,) or with a wild-type construct tagged with GFP (WT), or mock transfected (MT) for 24 h and 48 h, after which inhibition of VDR nuclear translocation was confirmed by an immunofluorescence assay. The results showed that after both 24 and 48 h, while the wild-type construct showed prominent nuclear localization, the localization of the VDR mutant was predominantly cytoplasmic (Fig 3B).

The antiviral activity of EB1089 towards DENV in the presence of the mutated VDR was evaluated through determination of the effects on virus titer. Cells were transfected with WT VDR or the mutated VDR or mock transfected, and after 24 h were infected with DENV 2, and then treated or not treated with EB1089. After a further 24 hours, the supernatant and cells were collected. The level of virus in the supernatant was determined by a plaque assay, while cells were again subjected to immunofluorescence analysis. The results showed that EB1089 treatment reduced titer by some 2–2.5 Log10 as compared to the untreated equivalent transfection/infections (Fig 3C). Markedly, while there were small (but significant) differences between the titers seen in the mock transfected/infected/treated cells and the transfected/infected/treated cells, there was no significant difference between the titers seen with the two VDR constructs (VDRm and WT) after EB1089 treatment (Fig 3C). Lastly, an immunofluorescence assay of the cells again showed that the localization of the WT VDR was predominantly nuclear (in both treated and untreated cells) while expression of the mutated VDR was predominantly cytoplasmic in both treated and untreated cells (Fig 3D and 3E). In addition, a marked reduction in DENV E protein was observed in all treated cells, as compared to untreated cells. In summary, no differences were seen in antiviral activity (as assessed by virus titer) between cells treated with EB1089 with or without inhibition of VDR nuclear localization.

### Effect of EB1089 on ZIKV-infected neural progenitor cells

**Fig 4:**
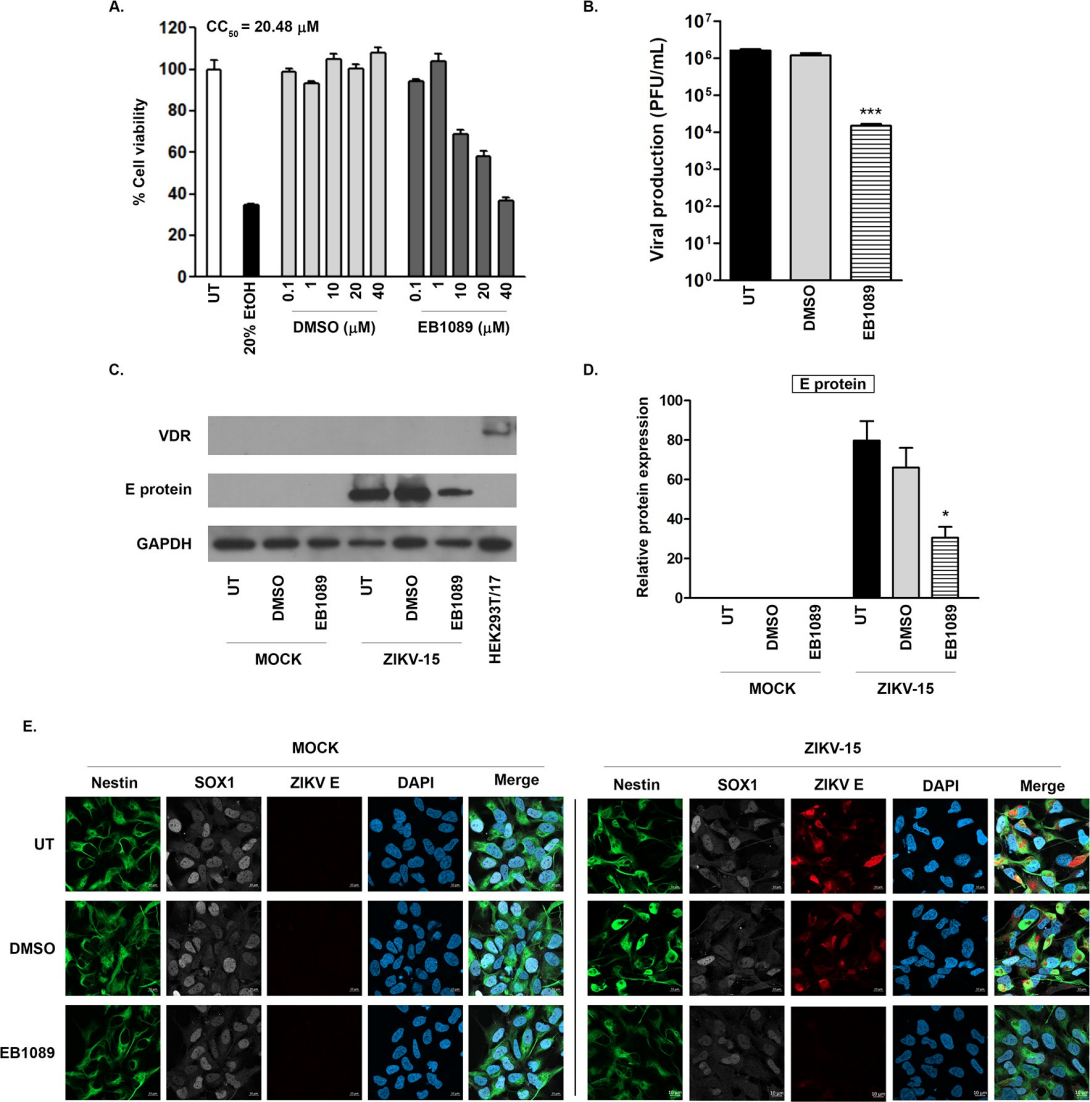
Viral inhibitory effect of EB1089 on ZIKV-infected neural progenitor cells. (A) Human neural progenitor cells (NPCs) derived from human induced pluripotent stem cells were cultured in the Matrigel-coated plates, followed by no treatment, treatment with 20% ethanol (EtOH), treatment with different concentrations of DMSO or EB1089 for 24 h, after which cell viability was determined by the MTT assay. The CC_50_ value of 20.48 μM was determined using the freeware ED50plus (v1.0) software (https://sciencegateway.org/protocols/cellbio/drug/data/ed50v10.xls). (B-D) NPCs were mock infected or infected with ZIKV and MOI 20 for 2h, after which the media was replaced with normal media (UT), media containing vehicle control (DMSO) or 10μM EB1089 for 24 h after which supernatant and cells were collected for analysis. (B) The level of infectious ZIKV was determined in the supernatant by plaque assay. (C) Expression of VDR and E protein and GAPDH were determined by western blotting. The relative protein expression levels were analyzed using Image J and signals for (D) E protein were determined and normalized with GAPDH. Protein extracted from uninfected HEK293T/17 cells used as a control for VDR expression. (E) The expression of nestin (green), SOX1 (white), and ZIKV E protein (red) were determined after staining with appropriate antibodies and examination under a confocal microscope. Nuclei were additionally stained with DAPI. All experiments were undertaken independently in triplicate with a duplicate plaque assay. Representative confocal images are shown. p-value; *< 0.05, **< 0.01, ***< 0.001 for significance compared with untransfected cells. Cropped images of western blots are shown, and full uncropped images can be found in the supplementary materials.

In a previous study, we established that a different, chemically synthesized vitamin D receptor agonist, ZD-6, had antiviral activity against ZIKV [26], both that study and this study were undertaken in HEK293T/17 cells. To investigate whether EB1089 has antiviral activity in a more biologically relevant system, the effect of EB1089 on ZIKV infected neural progenitor cells, which are one of the primary targets of this virus infection [50], was determined. Neural progenitor cells were differentiated from induced pluripotent stem cells via embryoid body formation, after which the cell stage was confirmed using the specific protein marker staining for Nestin, PAX6, SOX1, and Musashi-1, and negative cell stage controls including the pluripotent stem cell marker, Oct3/4 and the astrocyte marker, GFAP via an immunofluorescence assay (S1 Fig). After appropriate differentiation had been confirmed, the cytotoxicity of EB1089 towards these cells was determined. The results (Fig 4A) showed a CC50 value of 20.48 μM, in light of which a value of 10 μM was chosen to investigate the antiviral activity of EB1089. NPCs were cultured in a Matrigel-coated plate with a glass coverslip and infected with ZIKV at MOI 20 for 2 h before the removal of the virus. Subsequently, the infected cells were incubated with 10 μM EB1089 in parallel with untreated cells and a DMSO vehicle control. After 24 h of incubation, the number of infectious virions in the supernatant was determined by a plaque assay, and the infected cells were used to determine the levels of protein expression of ZIKV E protein and VDR. The results indicated that treatment of EB1089 decreased the virion production by approximately 2 log10 compared with the untreated and diluent control conditions (Fig 4B). Consistently, the levels of ZIKV E protein in infected NPCs treated with EB1089 showed a reduction in the western blot assay (Fig 4C and 4D), and the signal for ZIKV E protein was markedly reduced as compared to controls in an immunofluorescence assay (Fig 4E). Interestingly, VDR protein expression could not be detected in NPCs, while expression of this protein was readily detected in HEK293T/17 cells (Fig 4C).

## Discussion

The role of vitamin D in bone mineralization and calcium homeostasis has been well documented [17], but in addition to these roles, vitamin D and VDR have been shown to modulate processes including cell growth/ proliferation and immunomodulation, and an increasing number of studies have shown that vitamin D has anti-cancer and antiviral activity [18–20]. During the pandemic of COVID-19, studies showed that levels of serum vitamin D were lower in COVID-19 patients than in control groups (Hernandez, Jude) [40,51], while another study showed that in hospitalized COVID-19 patients, treatment with 25(OH)D3 (calcifediol) significantly reduced ICU admissions and mortality [52].

A similar association between low serum vitamin D levels and DHF/DSS was seen in a cohort of children in Sri Lanka, and vitamin D deficiency was more likely to be seen in patients than controls [53]. This was also shown in a study of vitamin D levels in adults with dengue in Singapore, where low vitamin D levels were associated with increased disease severity [54]. These results are consistent with a metabolomics study that identified a lower level of 1,25-dihydroxyvitamin D3 in DHF/DSS patients when compared to DF patients [55]. Supporting these studies, a small study of five DF patients reported that administration of oral calcium and vitamin D reduced the duration of signs and symptoms of DF [56]. In a similar study, Martinez-Moreno and colleagues showed that monocyte derived dendritic cells (MDDCs) from healthy donors who had been taking 4000 international units of vitamin D for 10 days were less susceptible to DENV infection than the same cells from volunteers who had received 1000 international units of vitamin D for 10 days [57]. However, in contrast to these studies, a study undertaken in Colombia showed that lower vitamin D levels were associated with decreased odds of progression of DF patients to DHF/DSS [58], while another study reported that vitamin D levels were higher in DF and DHF patients as compared to normal controls [59]. A third study on adults with non-severe or severe cases of dengue found vitamin D levels were elevated in both non-severe and severe cases as compared to healthy controls [60]. Thus, there remains no consensus on the association between vitamin D serostatus and either infection or severity.

The gene for VDR is located on chromosome 12q12-14 [61]. The gene for VDR is relatively large (>100 kbp), with two promotor regions, eight protein-coding exons (2–9) and six untranslated exons (1a-1f). Around 60 polymorphisms in the promoter, coding exons and 3’-untranslated region (3’-UTR) have been characterized [62,63]. A number of studies have looked for associations between polymorphisms in the VDR gene and dengue [59,60,64,65], as with studies investigating levels of vitamin D [53,55,56,58–60], there are inconsistencies within the literature. For example, while Loke and colleagues [64] found the less frequent “t” allele at position 352 of VDR (the TaqI polymorphism; rs731236), was associated with resistance to severe dengue, Singh and colleagues [65] found that the CC genotype at this position was found more frequently in dengue patients than in controls. Similarly, while Alagarasu and colleagues [59] found a significantly lower frequency of the ‘C’ allele of the rs7975232 polymorphism in dengue patients as compared to healthy controls, Singh and colleagues [65] found the GG genotype at this position to be higher in patients than controls. However, both studies by Alagarasu and colleagues [59] and Chakravarti and colleagues [60] showed that the “T” allele at rs2228570 was associated with severe dengue.

Thus, from patient-oriented studies, it is currently unclear both whether vitamin D exerts an antiviral effect and how it exerts this effect. However, a large number of in vitro studies have shown that vitamin D possesses antiviral or inhibitory activity against DENV [26,38–45], and one study previously showed activity of a vitamin D receptor agonist against ZIKV [26]. Several of these studies have shown that vitamin D primarily exerts its effect through VDR genomic effects which reduce the ability of cells to become infected [38,43,45], which would be consistent with the study of Mattinez-Moreno and colleagues on the reduced susceptibility of MDDCs from people taking a higher dose of vitamin D [57]. One of the possible mechanisms of vitamin D-mediated viral inhibitory action was the lowered expression of several essential virus entry receptors including C-type lectin mannose receptor (MR), toll-like receptors (TLRs) which further attenuated the production of inflammatory cytokines and type-I interferons antiviral response (IFN-I) [38,44,45,57,59]. In addition, vitamin D3 enhanced the suppressor of cytokine signaling, SOCS-1 via a miR-155-dependent mechanism [39,66]. However, somewhat surprisingly, the investigation of antiviral effect of vitamin D and vitamin D receptor agonists through VDR independent mechanisms remains poorly investigated.

In this study, we have shown that the antiviral activity of a vitamin D receptor agonist is perhaps exerted concomitantly between the VDR-genomic effects and the non-VDR mediated action. Neither upregulating, downregulating or retargeting VDR affected the magnitude of the antiviral effect of EB1089. Clearly, EB1089 does interact with VDR and downstream effects on gene transcription were shown, but our results support that this is not the only mechanism by which EB1089, and by extension vitamin D, predominantly exerts its antiviral activity. A previous study investigating the antiviral activity of vitamin D against HCV also proposed a non-VDR mediated mechanism of action [46], which was supported by an earlier study which suggested that the anti-HCV activity is mediated by the suppression of apolipoproteins [67]. The authors used CRISPR-Cas9 to knockout VDR from liver Huh7.5 cells, and found no impairment in the antiviral activity of vitamin D. Another study supporting non-VDR mediated viral inhibition showed that calcitriol enhanced the inhibitory effect of IFN-α on HCV infection via the upregulation of interferon-stimulated gene (ISGs) in VDR silenced Huh7.5 cells [34]. This effect was mediated through IFN-activated phosphorylated STAT-1 protein translocating into the nucleus after treatment with calcitriol. A recent study on macrophages treated with vitamin D3 also showed the activation of IFN-stimulated genes (ISGs) such as protein kinase R (PKR), 2’-5’-oligoadenylate synthetase 1 (OAS1) via an IFN-independent manner [40]. They proposed that vitamin D3 indirectly enhanced the activity of the JAK-STAT signaling pathway and downstream antiviral type I-IFN responses [68]. Other evidence to support a non-traditional genomic mechanism of action is a membrane VDR (VDRm), later identified as protein disulfide isomerase family A member 3 (PDIA3), has been reported to interact with both STAT and NF- κB which were able to cross the into the nucleus and regulate the expression of several genes including IFN-α and TNF-α [69–71]. A number of studies have shown that EB1089 can reduce cellular proliferation [72–74] and promote apoptosis [75,76]. Thus, the effects of EB1089 in reducing DENV replication could be a consequence of the slowed cell proliferation. However, this does not invalidate the observation that EB1089 is predominantly not working through VDR to exert its antiviral effects. We note that in some experiments there is an apparent mismatch between virus titer observed experimentally, and the levels of cellular DENV E protein see in western blot analysis. However, the two markers (virus titer and levl of E protein) cannot be used synonymously. Virus titer measures infectious virions only, and DENV replication as been shown to produce numerous not-infectious virions [77].

While infection with ZIKV is normally either asymptomatic, or results in a fairly mild, self-limiting disease, where infection occurs in a woman in the first or second trimester of pregnancy the virus can have significant impacts on the developing fetus [50]. While the fetus can suffer multiple developmental defects, the most serious impact is that on the developing fetal brain, and ZIKV infection in a fetus can result in the death of neural progenitor cells, in addition to a number of other types of cells in the brain (recently reviewed in [78]) resulting in severe microcephaly. Vitamin D can pass through the blood brain barrier [79], albeit in a restricted manner, and can additionally cross the placenta [80]. Thus, this study additionally looked at the antiviral activity of EB1089 in a more biologically relevant model, namely ZIKV infection of neural progenitor cells, differentiated from hiPSCs. The results showed a nearly 2Log10 reduction of virus titer in response to EB1089 treatment, and a marked reduction of ZIKV E protein. Interestingly, VDR expression was not detected in these cells by western blotting, giving further support for EB1089 exerting its antiviral effect independently of VDR.

## Conclusion

This study has presented evidence that the vitamin D receptor agonist EB1089 can exert its antiviral effect independently of the VDR. Neither upregulation, downregulation, or retargeting of VDR affected the magnitude of the EB1089 antiviral effect. We have additionally shown that EB1089 can inhibit ZIKV infection of neural progenitor cells, suggesting that this might have clinical applications in areas where ZIKV transmission is ongoing.

## Supporting information

S1 Fig Human induced pluripotent stem cells (hiPSCs)-derived neural progenitor cells (NPCs) differentiation. (PDF)

S1 Table List of Primary antibodies used for immunofluorescence and western blot studies. (PDF)

S2 Table List of Secondary antibodies used for immunofluorescence and western blot studies; uncropped wester blots. (PDF)

S1 File (PDF)
