# Supplementary material for: The vitamin D receptor agonist EB1089 can exert its antiviral activity independently of the vitamin D receptor
Source: PLoS One. 2023 Oct 17;18(10):e0293010. doi: 10.1371/journal.pone.0293010 (PMC10581485; doi:10.1371/journal.pone.0293010)
Supplement: S1 Fig — (PDF) [file pone.0293010.s001.pdf]

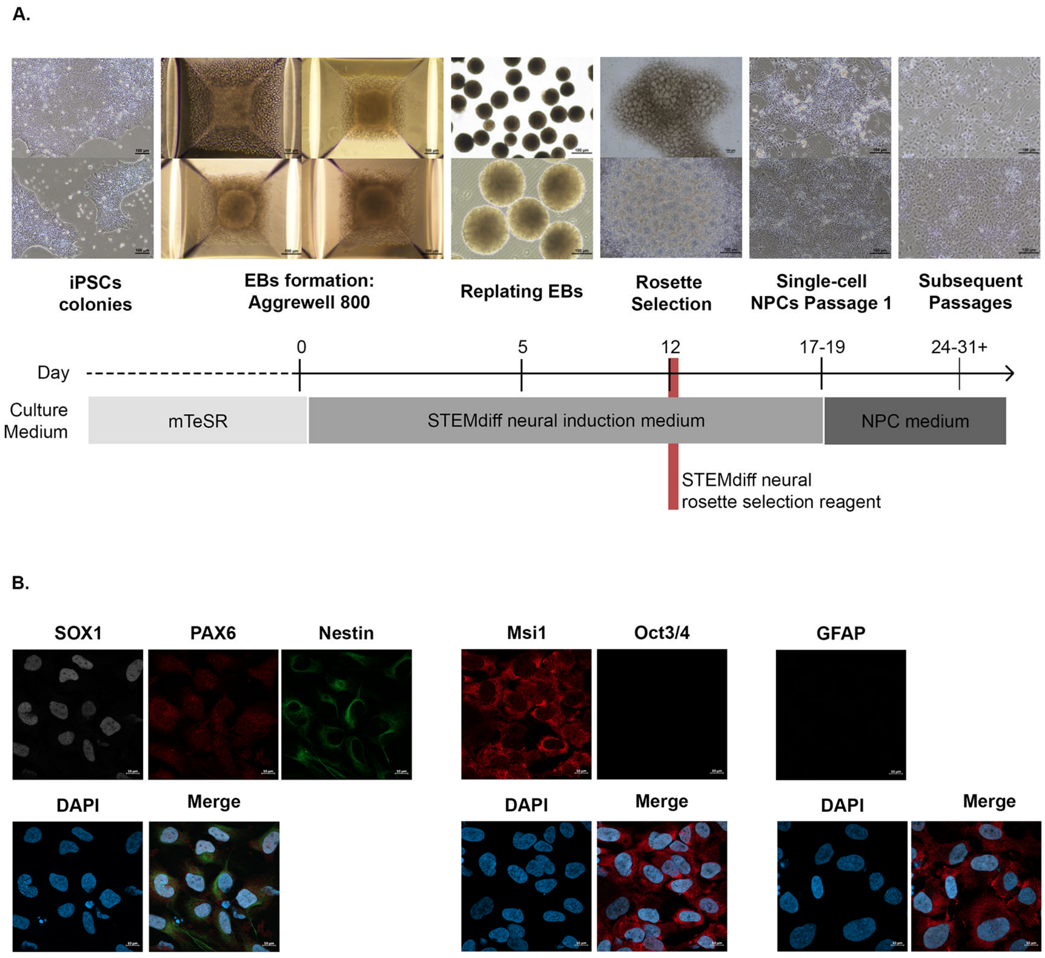

Supplementary figure S1. Human induced pluripotent stem cells (hiPSCs)-derived neural progenitor cells (NPCs) differentiation

The NPCs differentiation protocol was performed using the STEMdiff SMADi Neural Induction Kit as shown in schematic A. Briefly, human-induced pluripotent stem cells (hiPSCs) were cultured as the colonies clustered with mTeSR medium on a Matrigel-coated plate. Subsequently, the generation of EBs was performed in an Aggrewell 800 using STEMdiff Neural Induction Medium and the medium was partially changed for 5 days. Then, the EBs were harvested from the individual well and replated on a Matrigel-coated 6-well plate. During full medium change for up to 7 days, the rosette form of NPCs was selected using STEMdiff neural rosette selection reagent and replated into a new Matrigel-coated plate. Then, the first passage of NPCs was accessible using the accutase enzyme to dislodge the cells and resuspend to be a single -NPCs. The NPCs were subsequently cultured in either the 6-well plate or flask cultured and maintained in an NPC medium. Finally, hiPSCs-derived NPCs were confirmed for the cell stage with the NPC marker staining using an immunofluorescence assay including PAX6, nestin, Musashi 1and SOX1 and the negative stage iPSCs and astrocyte markers Oct3/4 and GFAP, respectively (B). All experiments were performed in triplicate.
