# Supplementary material for: The vitamin D receptor agonist EB1089 can exert its antiviral activity independently of the vitamin D receptor
Source: PLoS One. 2023 Oct 17;18(10):e0293010. doi: 10.1371/journal.pone.0293010 (PMC10581485; doi:10.1371/journal.pone.0293010)
Supplement: S1 Table — (PDF) [file pone.0293010.s002.pdf]

Supplementary Table S1. List of Primary antibodies used for immunofluorescence and western blot studies

Species	Antibody name	Dilution		Catalog No.	Company
Mouse mAb	Dengue NS5 antibody	WB	1:3000	GTX629446	GeneTex, Irvine, CA
Mouse mAb	Dengue Virus Type 1-4 Antibody (D1-11(3))	WB<br>IF	1:1000<br>1:50	MA1-27093	Thermo Fisher Scientific Inc., Waltham, MA
Mouse mAb	GAPDH antibody	WB	1:10000	SC-32233	Santa Cruz Biotechnology Inc., Dallas, TX
Mouse mAb	GFAP Monoclonal Antibody (ASTRO6)	IF	1:25	MA5-12023	Thermo Fisher Scientific Inc., Waltham, MA
Mouse mAb	Nestin Antibody Clone 10C2	IF	1:500	60091	Stemcell Technologies, Vancouver, Canada
Mouse mAb	Oct3/4 (C-10)	IF	1:50	sc-5279	Santa Cruz Biotechnology Inc., Dallas, TX
Rabbit pAb	PAX-6 Antibody	IF	1:50	60094	Stemcell Technologies, Vancouver, Canada
Rabbit mAb	Recombinant Anti-Musachi 1 / Msi1 antibody	IF	1:500	EP1302	Abcam, Cambridge, United Kingdom
Goat pAb	SOX1 Antibody	IF	1:50	AF3369	R&D Systems, Minneapolis, MN
Mouse mAb	VDR (D-6) antibody	WB	1:500	SC-13133	Santa Cruz Biotechnology Inc., Dallas, TX
Rabbit pAb	Zika virus Envelope protein antibody	WB<br>IF	1:5000<br>1:50	GTX-133314	GeneTex, Inc, Irvine, CA
