# Supplementary material for: The vitamin D receptor agonist EB1089 can exert its antiviral activity independently of the vitamin D receptor
Source: PLoS One. 2023 Oct 17;18(10):e0293010. doi: 10.1371/journal.pone.0293010 (PMC10581485; doi:10.1371/journal.pone.0293010)
Supplement: S2 Table — (PDF) [file pone.0293010.s003.pdf]

Supplementary Table S2. List of Secondary antibodies used for immunofluorescence and western blot studies.

Species	Antibody name	Dilution		Catalog No.	Company
Donkey pAb	Anti-goat Alexa Fluor 568	IF	1:100	A11057	Thermo Fisher Scientific Inc., Waltham, MA
Goat pAb	Anti-human Alexa Fluor 647	IF	1:100	A21445	Thermo Fisher Scientific Inc., Waltham, MA
Donkey pAb	Anti-mouse Alexa Fluor 488	IF	1:100	A21202	Thermo Fisher Scientific Inc., Waltham, MA
Donkey pAb	Anti-rabbit Alexa Fluor 647	IF	1:100	A31573	Thermo Fisher Scientific Inc., Waltham, MA
Goat pAb	HRP-conjugated anti-mouse IgG	WB	1:5000	31430	Thermo Fisher Scientific Inc., Waltham, MA
Goat pAb	HRP-conjugated anti-rabbit IgG	WB	1:5000	31460	Thermo Fisher Scientific Inc., Waltham, MA
