# Supplementary material for: The vitamin D receptor agonist EB1089 can exert its antiviral activity independently of the vitamin D receptor
Source: PLoS One. 2023 Oct 17;18(10):e0293010. doi: 10.1371/journal.pone.0293010 (PMC10581485; doi:10.1371/journal.pone.0293010)
Supplement: S1 File — (PDF) [file pone.0293010.s004.pdf]

Figure 1B VDR overexpresion

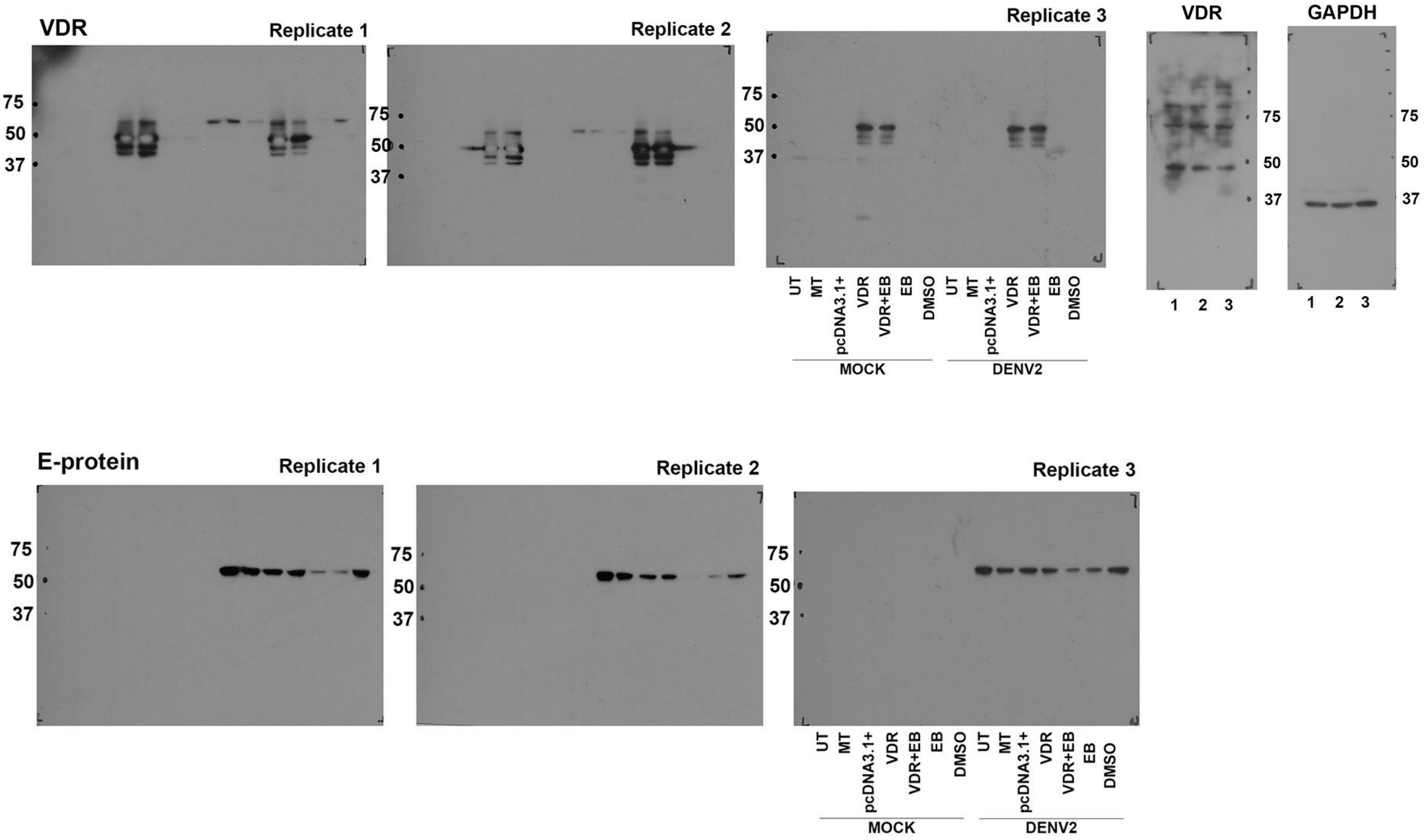

Figure 1B

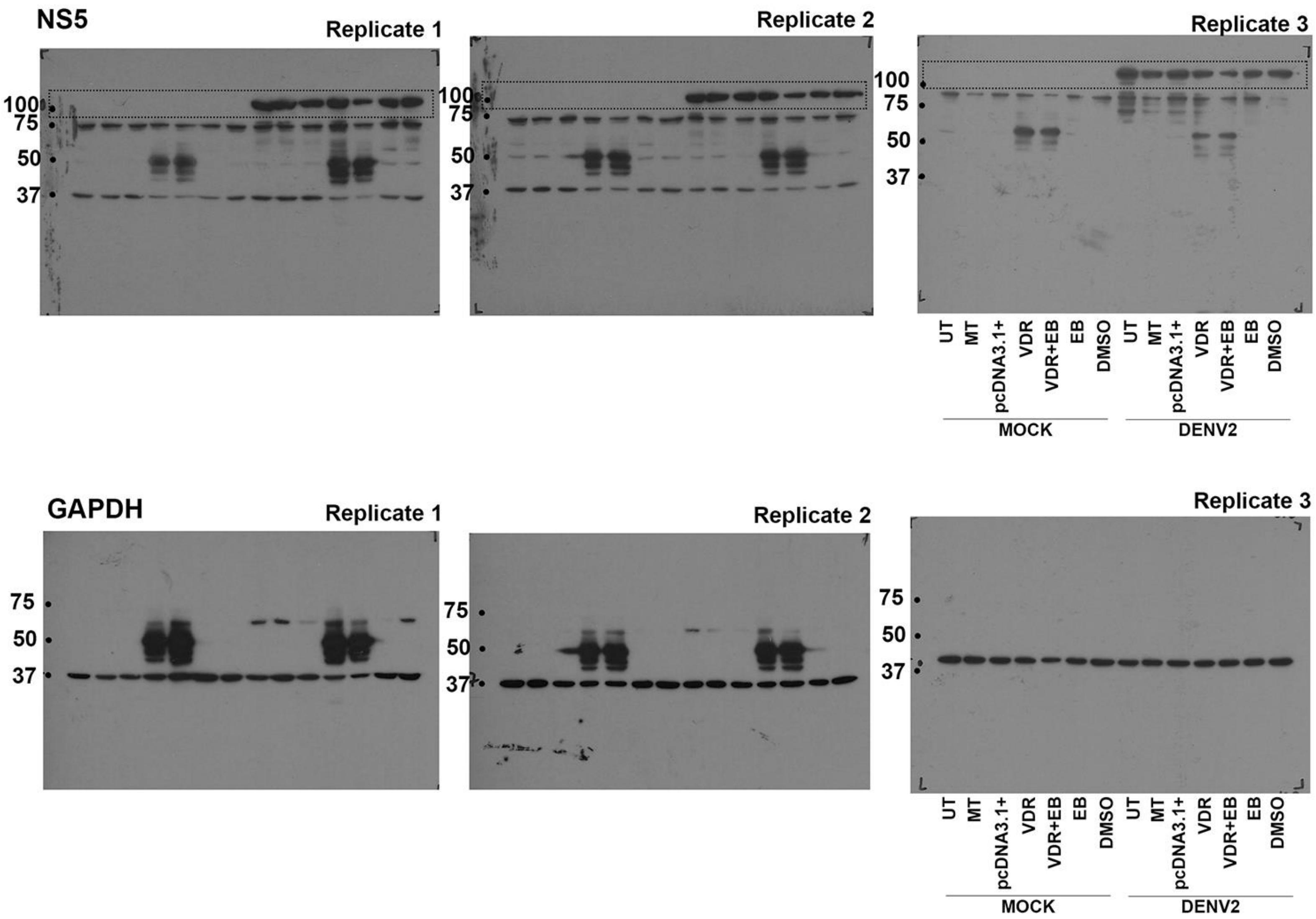
NS5
Replicate 1
Replicate 2
Replicate 3
100
75
50
37
UT
MT
pcDNA3.1+
VDR
VDR+EB
EB
DMSO
MOCK
DENV2
GAPDH
Replicate 1
Replicate 2
Replicate 3
75
50
37
UT
MT
pcDNA3.1+
VDR
VDR+EB
EB
DMSO
MOCK
DENV2

Figure 2A: VDR

Figure 2A: GAPDH

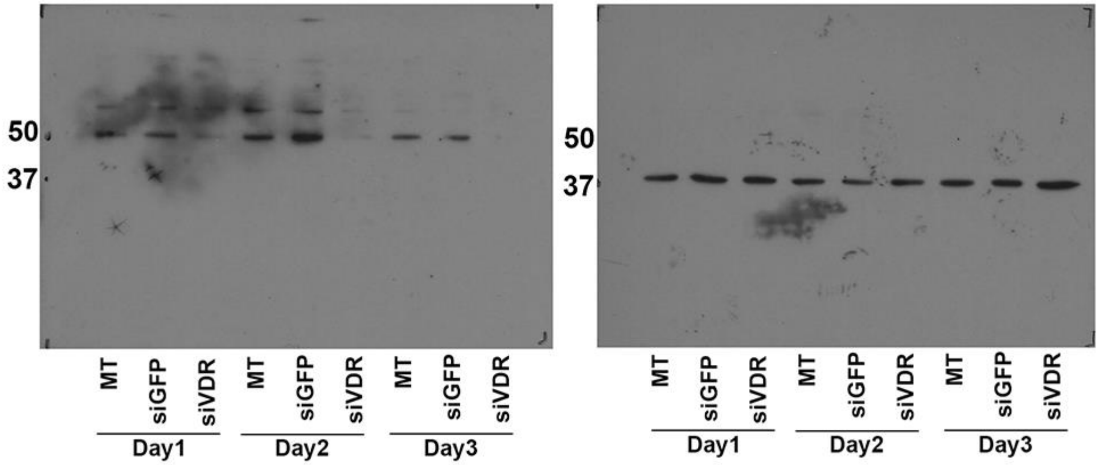

Figure 2C

VDR

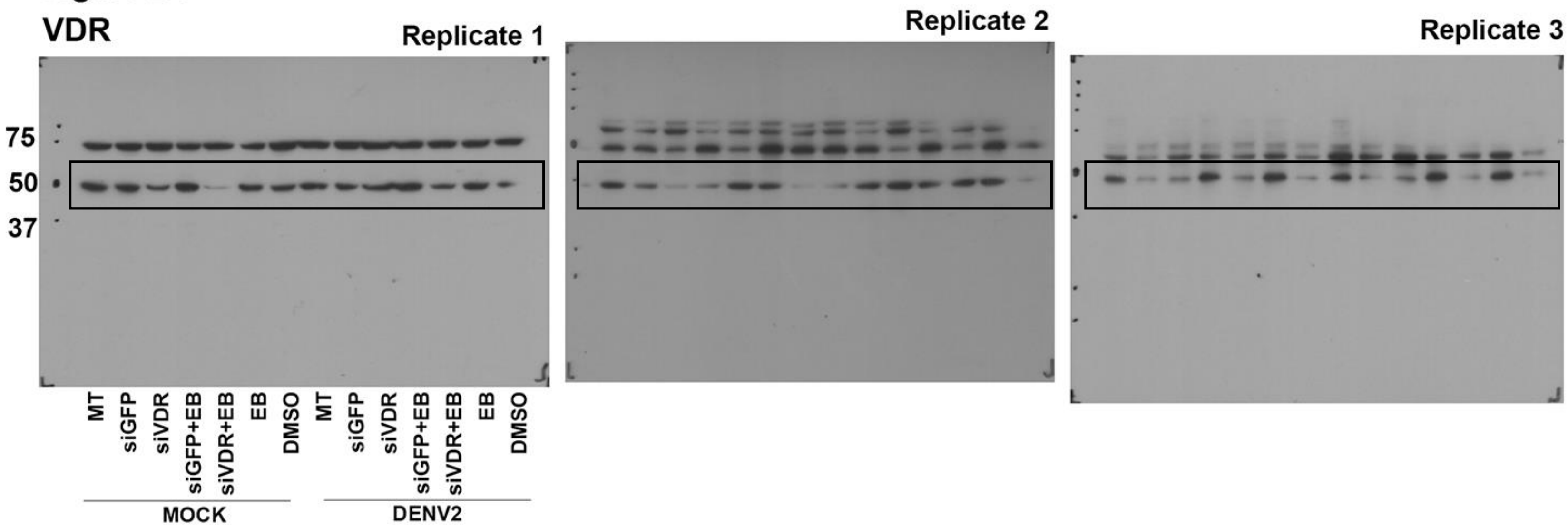

Figure 2C

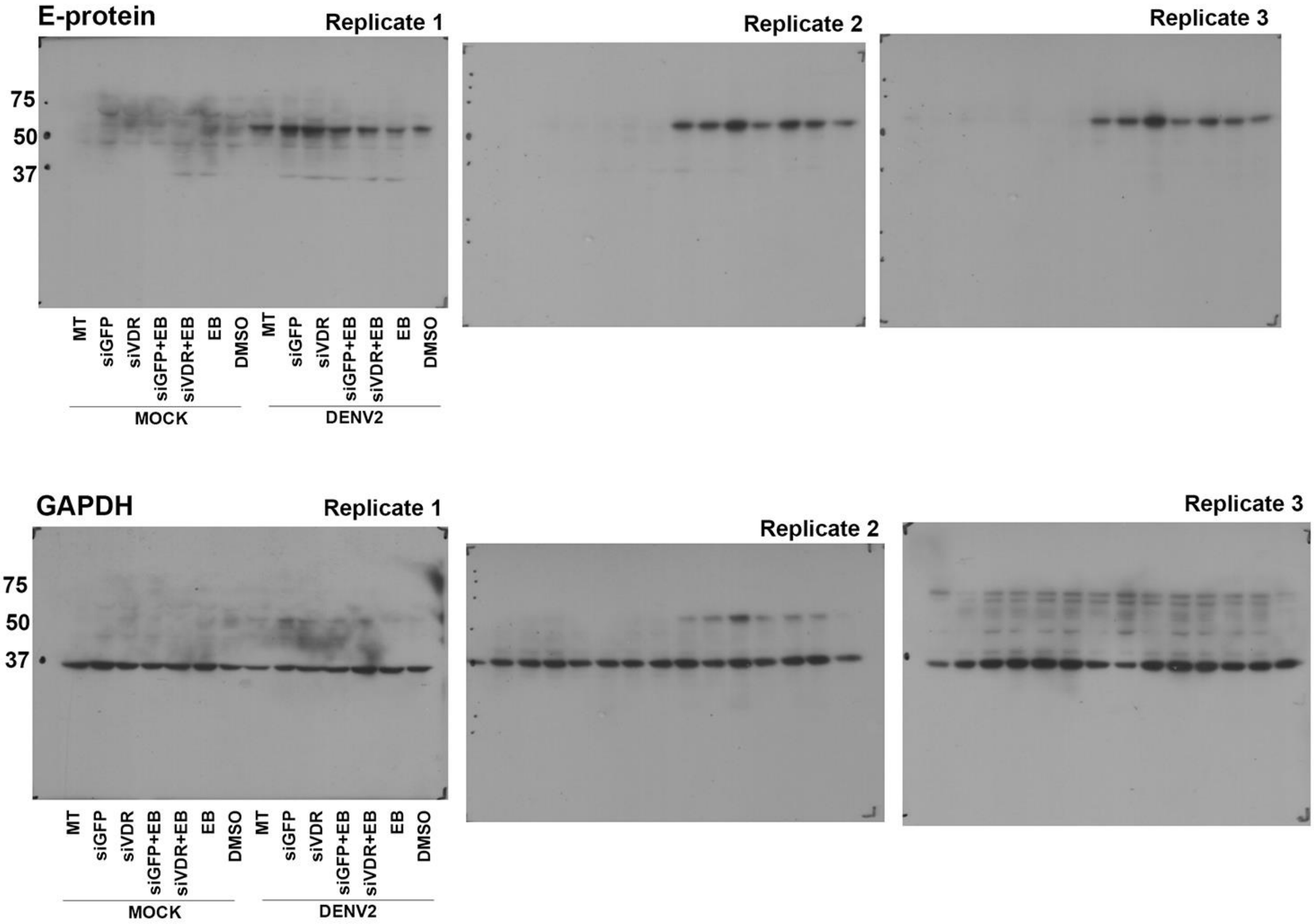
E-protein
Replicate 1
Replicate 2
Replicate 3
75
50
37
MT
siGFP
siVDR
siGFP+EB
siVDR+EB
EB
DMSO
MT
siGFP
siVDR
siGFP+EB
siVDR+EB
EB
DMSO
MOCK
DENV2
GAPDH
Replicate 1
Replicate 2
Replicate 3
75
50
37
MT
siGFP
siVDR
siGFP+EB
siVDR+EB
EB
DMSO
MT
siGFP
siVDR
siGFP+EB
siVDR+EB
EB
DMSO
MOCK
DENV2

Figure 4B

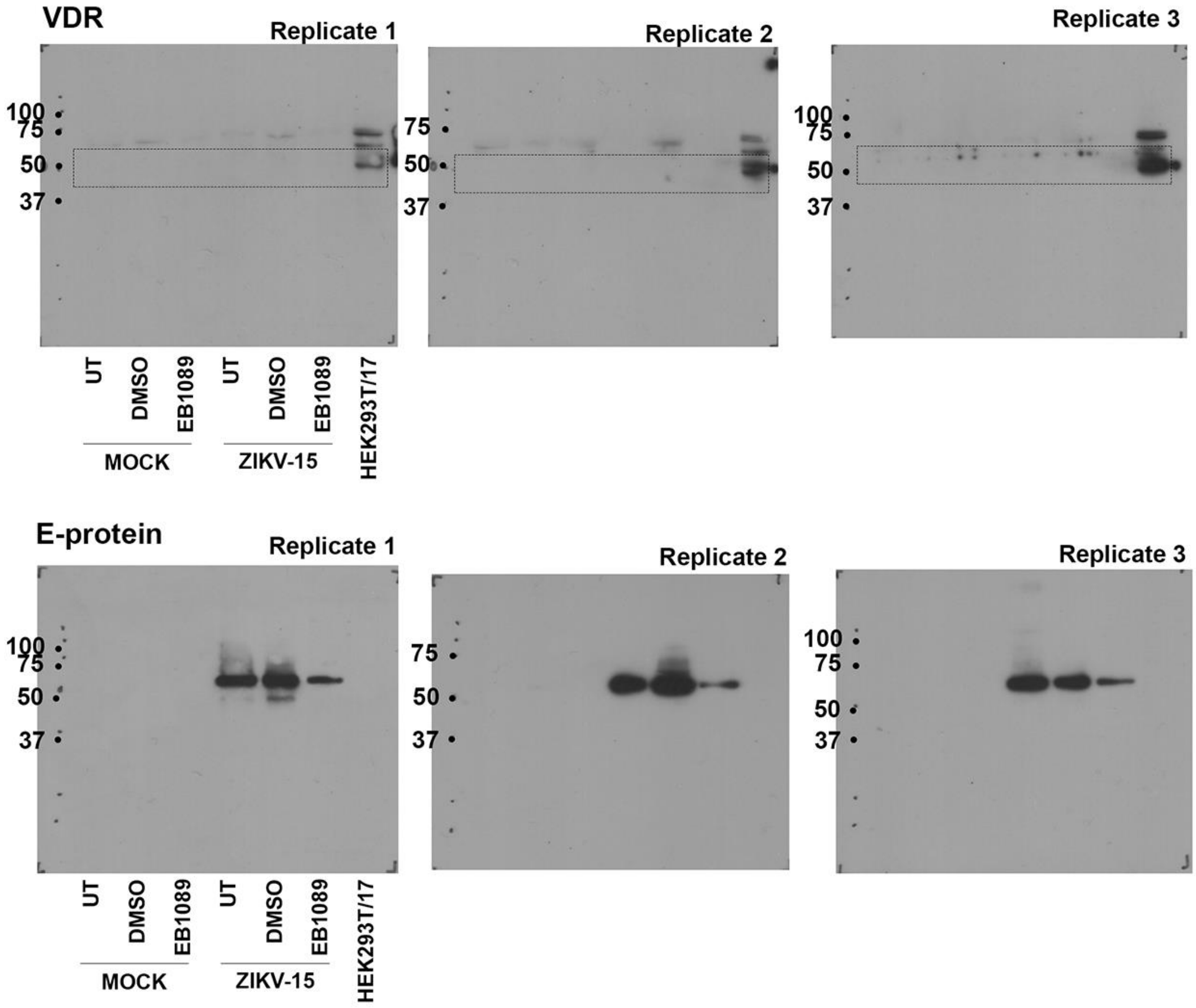
VDR
Replicate 1
Replicate 2
Replicate 3
100
75
50
37
UT
DMSO
EB1089
UT
DMSO
EB1089
HEK293T/17
MOCK
ZIKV-15
E-protein
Replicate 1
Replicate 2
Replicate 3
100
75
50
37
UT
DMSO
EB1089
UT
DMSO
EB1089
HEK293T/17
MOCK
ZIKV-15

Figure 4B

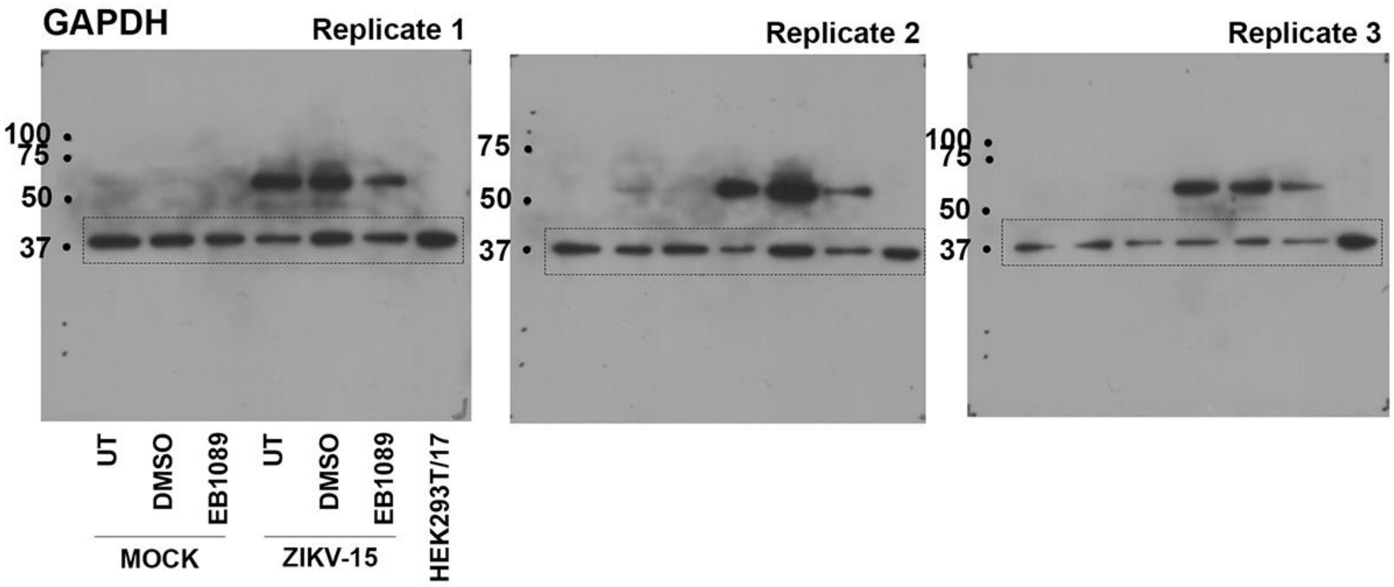
GAPDH
Replicate 1
Replicate 2
Replicate 3
100
75
50
37
75
50
37
100
75
50
37
UT
DMSO
EB1089
UT
DMSO
EB1089
HEK293T/17
MOCK
ZIKV-15
